# Hemorrhage from the Extraction of Teeth

**Published:** 1858-09

**Authors:** F. D. Thurman

**Affiliations:** Dentist, Atlanta, Geo.


### ARTICLE IV.

*Hemorrhage from the Extraction of Teeth.* By F. D. THURMAN, Dentist, Atlanta, Ga.

Alarming cases of hemorrhage from the extraction of teeth are of such rare occurrence, that the subject loses much of its importance. Though as we do occasionally read of a case proving fatal, it is important that all who extract teeth, should be informed as to the proper treatment under such exigencies.

Occasionally persons are met with, who manifest the hemorrhagic diathesis as an organic idiosyncrasy.

In such cases, the slightest wounds of any kind are exceedingly dangerous, as the blood is deprived of its coagulable property, and will very likely continue to flow until it is arrested by mechanical or other means.

TREATMENT.—In simple cases, all that is generally necessary is simply to plug up the cavities from which the roots were extracted with a little cotton wool dipped into the oil of turpentine, tannic acid, or dilute nitric acid. But as the blood in some cases is so exceedingly thin that it passes readily through the cotton, it becomes necessary to resort to plugs of a more solid nature, such as a strip of cloth rolled into a conical form, and dipped into melted wax, or what is incomparatively better, a piece of gutta percha softened in warm water, then pressed firmly into the cavities.

In nearly every case one or another of these remedies will succeed.

Though in some rare instances all of these and numerous others have failed. But if the following plan has ever proved unsccessful, it has never been published. It is a plan which is now practiced by nearly all skillful Dental Surgeons, in extreme cases, to wit:

Take a perfect impression of the mouth in softened wax, get up metalic dies in the usual way, and strike up a plate of tin, silver, or gold, so as to fit the parts perfectly for some distance around the bleeding points, and confine it down with clasps or otherwise. If properly done, so that the plate presses equally on every part, it is utterly impossible for the blood to escape.

